# Impact of scion/rootstock reciprocal effects on metabolomics of fruit juice and phloem sap in grafted *Citrus reticulata*

**DOI:** 10.1371/journal.pone.0227192

**Published:** 2020-01-10

**Authors:** Zipora Tietel, Snehil Srivastava, Aaron Fait, Noemi Tel-Zur, Nir Carmi, Eran Raveh

**Affiliations:** 1 Agricultural Research Organization, Gilat Research Center, Gilat, Israel; 2 French Associates Institutes for Agriculture and Biotechnology of Drylands, J. Blaustein Institutes for Desert Research, Ben-Gurion University of the Negev (BGU), Sede-Boqer Campus, Sede Boker, Israel; Institute of Mediterranean Forest Ecosystems of Athens, GREECE

## Abstract

**Background:**

Rootstock has a significant impact on plant growth and development, including fruit maturation. However, the existence of mutual interaction between scion and rootstock is often neglected. To explore the origin of different fruit quality traits in citrus, we studied the effect of rootstock and the reciprocal interaction between scion and rootstock of nine combinations; three mandarin varieties grafted on three different rootstocks. We analyzed the metabolic profile of juice via gas and liquid chromatography-mass spectrometry (GC-MS and LC-MS, respectively). Additionally, we profiled phloem sap composition in the scion and the rootstock. Quality traits of fruit and their physio-chemical characteristics were also evaluated.

**Results:**

For all three cultivars, rootstock was found to affect fruit yield and biochemical fruit quality parameters (sugar and acidity) in interactions with the scions. In mandarin juice, eight of 48 compounds (two primary and six secondary) were related directly to the rootstock, and another seven (one primary and six secondary) were interactively affected by scion and rootstock. In scion and rootstock sap, six and 14 of 53 and 55 primary metabolites, respectively, were directly affected by the rootstock, while 42 and 33 were affected by rootstock interactively with scion, respectively.

**Conclusion:**

In this work, we show for the first time a reciprocal effect between rootstock and scion. Based on our results, the scion and rootstock interaction might be organ, distance or time dependent.

## Introduction

Grafting is a well-developed technique in fruit trees, which combines a scion and a rootstock to form a new plant with a blend of characteristics [1]. Several studies on apples, citrus and other deciduous fruit trees have reported the relationships between the type of rootstock and scion in a grafted tree is based on various physiological parameters [2–5]. Selection of an appropriate graft combination can be pivotal in respect to nutrient uptake, water potential, plant vigor, fruit quality and yield efficiency [6–9]. Several research reports have documented the significant impact of rootstock on fruit quality in grafted cultivars [4,10–12]. However, there is no available information regarding the direct effects of rootstock on scion physiology and fruit composition, and the interactive effects of scion/rootstock combinations.

Citrus cultivars are widely commercialized and consumed worldwide, with oranges and mandarins being the most important species [13]. Mandarins are rich in primary metabolites such as organic acids, sugars and amino acids. They also present a great diversity of valuable secondary metabolites such as carotenoids, polyphenols (mainly flavonoids: flavanones and flavones, phenolic acids and limonoids), in addition to volatile compounds that contribute to their nutritional characteristics and health benefits [10,14]. The effect of rootstocks on fruit yield is usually related to their impact on fruit size and quality; i.e., sugar and acid contents, sugar: acid ratio and antioxidant potential [4]. For example, one of the most commonly used rootstocks, Sour Orange (SO) (*Citrus aurantium* L.) is known to produce moderate yields of orange and grapefruit with average fruit size and good quality [15]. SO rootstock enhances tolerance to salinity and alkalinity but on the other hand, is susceptible to citrus nematode and Citrus Tristeza Virus (CTV). In comparison to SO, Volkameriana lemon (Volka) (*C*. *volkameriana*, Ten and Pasq.) rootstock provides higher growth vigor, fruit size and total yield. This rootstock is tolerant to *Phytophthora* and CTV but its fruit quality is lower.[16] Similarly, SB-812 [*C*. *sunki* (Hort. ex Tan.) × *Ponciru strifoliata* (L.)] rootstock gives good-quality fruit and exhibits tolerance to CTV and citrus blight, but is salt-sensitive [4].

Crosstalk between the above and below graft parts is conducted by plant vascular systems, the xylem and phloem. Phloem sap is rich in nutrients, containing a complex mixture of organic and inorganic substances, especially sugars, amino acids, organic acids, minerals and hormones [17,18]. Sugars and amino acids are the predominant metabolites in phloem sap, but their content varies in different species [17]. Tree growth mainly depends on the efficient and controlled distribution of organic and inorganic substances across the tree parts [19]. Long-distance signaling in response to stress and resource levels are also delivered via the vascular system, for example, sugars and polyols are repartitioned from the leaves to the roots for storage, and ABA and GABA are delivered to the shoots to regulate stomata conductance and signal changes in nitrogen allocation [20]. Despite the importance of scion-rootstock combination for commercial marketing of citrus fruits, relatively little is known regarding the mechanisms involved in the crosstalk between these two plant organs, which involves the forced interaction of two different genotypes. Studies exploring scion-rootstock interaction focus primarily on the effect of tree vigor and pest tolerance. For example, in grapevines, rootstock might have an impact on scion vigor, nutrient uptake [21,22] and production of secondary metabolites in xylem and phloem sap, as host defense against pathogens [23].

Most of the recent studies examine the effect(s) of rootstock on scion development; however, the information regarding the effect of scion on rootstock is lacking. In this work, we explored the reciprocal interaction between scion and rootstock, tackling the role of the phloem sap composition on the metabolite profile of juice, and the physio-chemical characteristics of fruit using gas and liquid chromatography-mass spectrometry in different citrus cultivars.

## Materials and methods

### Experimental orchard

The study was carried out in an experimental orchard established in 2005, in the western Negev in Israel (Gilat Research Center; 31°19’28.15” N, 34°39’20.29” E), 120 m above sea level. The semi-arid Mediterranean climate here is hot and dry during the summer (May-August) and rainy during the winter (November–March). Precipitation in this location ranges from 150 to 350 mm per year, with an average of 280 mm per year [4]. The terrain is flat, and the soil is sandy loess with pH > 8.0 [4].

Based on regional recommendations, the orchard was fertigated twice a week with 75 mg L^-1^ NH_4_NO_3_ applied between April and October via four 3.7 L h^-1^ drippers per tree [4]. In addition, iron chelate (FeEDDHA, SequestreneFe-138, Ciba Geigy, Basel, Switzerland) was applied twice a year through the fertigation system [4].

### Scion/Rootstock combinations

**Fig 1:**
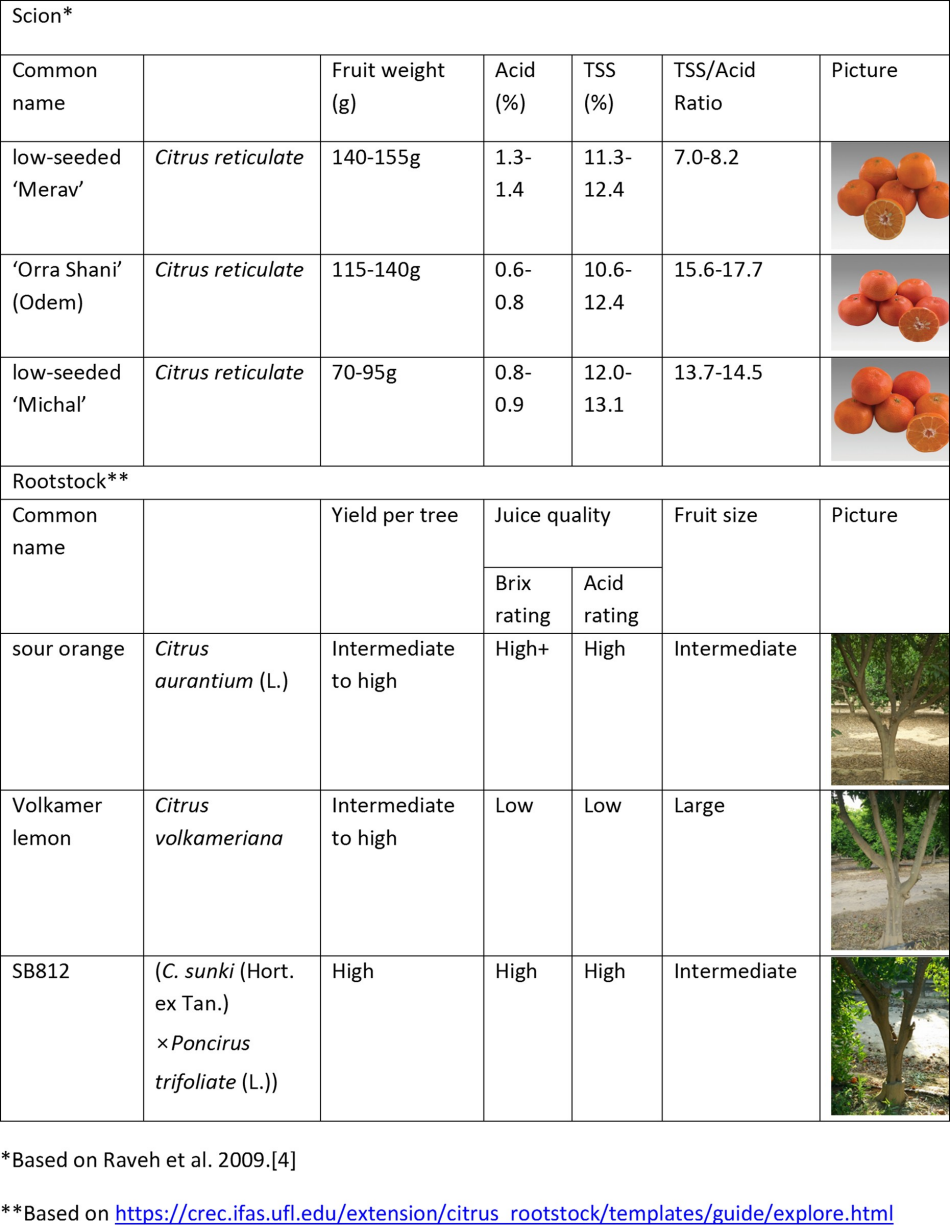
Citrus scion and rootstock general characteristics.

Trees were planted in five randomized blocks of four trees (spaced 3 x 6 m) per block of the examined scion/rootstock combinations. The orchard comprises nine scion/rootstock combinations of mandarin. Three different easy-peeler *Citrus reticulate* mandarins were the cultivars: 'Orra Shani', 'Merav' and 'Michal', which were grafted onto three different rootstocks: Sour orange (designated SO) [*C*. *aurantium* (L.)], Volkameriana lemon (designated Volka) [*C*. *volkameriana* (Ten. and Parq)] and SB-812 (designated 812) [*C*. *sunki* (Hort. ex Tan.) × *Poncirus trifoliate* (L.)]. Fig 1 presents typical characteristics of the above varieties, including fruit weight and yield, as well as fruit physio-chemical quality parameters.

### Fruit yield

Fruit yield data were collected from full-grown trees for three successive years, 2008, 2009 and 2010. The total fruit yield from each tree (four trees in each of the five blocks; twenty trees for each rootstock/scion combination) was weighed separately, and the effect of scion/rootstock interaction on average accumulated yield per tree was calculated.

### Juice sample preparation

The fruit was harvested at full maturity, at the peak of the commercial harvest season (December 2014). Fifty fruit from each of the nine combinations were randomly sampled (two replicates of five fruit from each of the five blocks; and the juice was immediately squeezed. Ten ml from each replicate was stored at -20°C for TSS (%), acidity (%), and ascorbic acid (100 mg/ml) analyses, and about 40 ml was stored at -80°C for gas chromatography-mass spectrometry (GC-MS) and ultra-performance liquid chromatography (UPLC-MS) analysis.

### Juice total soluble sugar, acid and ascorbic acid content

Total soluble sugar content in the juice was determined with a digital Brix refractometer [Milwaukee, MA871]. Acid content was assessed by standardization of NaOH using phenolphthalein as an indicator by titration method. Vitamin C (ascorbic acid) was tested with DCPIP (2, 6- dichlorophenol indophenols) as described by Raveh et al [4].

### Extraction of juice metabolites for profiling

Metabolic profiling was carried out using a GC-MS based approach [24]. The juice samples stored at -80°C were freeze-dried. Then the samples were homogenized under liquid nitrogen using mortar and pestle, and 20 mg of freeze-dried juice samples were further ground to powder form under liquid nitrogen using RETCH-mill with pre-chilled holders and two grinding beads for 4 min at 15Hz. Extraction was done according to Fiehn and colleagues for GC-MS [25]. One hundred μl of pre-chilled 100% methanol was then added to the frozen powder. The metabolites were then extracted by adding 1 ml of extraction mixture (methanol:chloroform: water; 2.5:1:1 v/v/v). Internal standards, i.e., 0.2 mg/ml ribitol in water, 1 mg/ml ampicillin in water, 1 mg/ml corticosterone in methanol and 5 mg/ml heptadecanoic acid in chloroform, were subsequently added. The samples were incubated for 10 min at 4°C on an orbital shaker followed by incubation for another 10 min in an ultrasonication bath at room temperature, in order to release the cell content. The samples were centrifuged in an ultracentrifuge (Centrifuge 5417 R), to separate metabolites from cell debris, at 14,000 rpm. Polar metabolites were separated from non-polar metabolites by adding to the samples 300 μl of UPLC grade water and 300 μl of chloroform. Following second centrifugation of 2 min at 14,000 rpm, the upper methanol/water phase containing the polar metabolites was separated from the lower chloroform phase containing the nonpolar metabolites. Thirty μl of the upper phase were used for GC-MS analysis, and another 70 μl were stored in -80°C for UPLC-MS analysis. The 30 μl samples were reduced to dryness using a vacufuge. Dried extracts were derivatized according to the established protocol [24] for GC-MS analysis with standard alkane mixture (0.029% v/v *n*-dodecane, *n*-pentadecane, *n*-nonadecane, *n*-docosane, *n*-octacosane, *n*-dotracontane, and *n*-hexatriacontane dissolved in pyridine (0.0075% H_2_O) (Sigma–Aldrich, Jerusalem, Israel) and *N*-methyl-*N*-[trimethylsilyl] trifluoroacetamide (MSTFA, Macherey-Nagel GmbH and Co. KG, Düren, Germany).

### Analysis of juice polar primary metabolites using GC-MS

For GC-MS analysis, one μL of the derivatized sample was injected in splitless mode onto a 30-m VF-5ms GC column with 0.25 mm i.d., a film thickness of 0.25 μm, and +10 m EZ-Guard (Agilent). The GCMS system consisted of an AS 3000 autosampler, a Trace GC Ultra gas chromatograph, and a DSQII quadrupole mass spectrometer (Thermo-Fisher ltd). The parameters of the machine were as previously described [26]. Annotation was done by the National Institute of Standards and Technology (NIST, Gaithersburg, MD, USA) algorithm incorporated in the Xcalibur^®^ data software (version 2.0.7) against RI libraries from the Max-Planck Institute for Plant Physiology in Golm, Germany (http://www.mpimp-golm.mpg.de/mms-library/) and finally normalized by respective ribitol amount. Results were elaborated and analyzed by multivariate data analysis approaches using the software package MetaboAnalyst 3.0 [27].

### Analysis of juice secondary polar metabolites using UPLC-QTOF-ESI

The extraction of metabolites was described above, as a combined GC-MS/LC-MS extraction protocol was used [28]. After extraction, the samples were filtered through an HPLC certified Acrodisc CR 13 mm syringe filter with a 0.2 μm PTFE membrane. The separation was carried out on a Waters Xevo ultra-performance liquid chromatography (UPLC) with a mass detector quadrupole time-of-flight (QTOF) analyzer equipped with electrospray ionization (ESI) in both positive and negative modes. The mobile phase consisted of 95% water: 5% acetonitrile: 0.1% formic acid (phase A) and 0.1% formic acid in acetonitrile (phase B). The solvent gradient program was the following: 100–60% solvent A over the first 8 min, 60–0% solvent A over 1 min and returned to the initial 100% solvent A in 3.5 min. Acquired chromatograms, mass spectra, metabolite marker tables, and statistical plots were evaluated using MassLynx software, version 4.1. The mass fragmentation of the elucidated metabolites was compared with the ChemSpider (http://www.chemspider.com) and Metlin (https://metlin.scripps.edu) online databases. The quantification of the compounds was based on the relative peak response area of each mass signal after Pareto scaling in the chromatograms and normalized to the internal standard ampicillin [29].

### Phloem sap collection

A strip (about 5 by 7cm) of phloem tissue (phloem and its adjacent bark) was removed using a chisel from scion and rootstock parts (10 cm above and below the grafting zone) in the second week of August 2015. The tissue removal was done in the summer season because in this period the cambium tissues are active, allowing a precise separation between the xylem and the phloem parts. The phloem strips were cut into 1–1.5 cm pieces, and 3 to 5 pieces were placed vertically in a 0.5 ml tube. A small hole was made at the bottom of the tube, which was placed into a 2 ml tube for centrifugation. [30] After centrifuged at 12,000 rpm for 8 min, the phloem sap was collected and stored at -80°C until analysis. Three replicates from each of the nine scion/rootstock combinations (27 scion sap samples and 27 rootstock sap samples) were used for the GC-MS analysis.

### Extraction of phloem sap primary metabolites for GC-MS metabolic profiling

Metabolic profiling was carried out using a GC-MS based approach. Fifty μl of sap samples were used to perform the extraction process prior to the GC-MS run. Extraction was done using the protocol described in juice analysis previously. Extracted sap sample, 200 μl, was reduced to dryness in a vacufuge. The derivatization process was carried out in the same way as it was performed for juice, as mentioned before.

### Statistical analysis

Fruit yield and quality statistical analyses were done by Tukey test (*P* < 0.05) using JMP 8 and Prism program, version 5.0. Comparisons among the combinations based on the scion/rootstock reciprocal interaction on fruit quality and phloem sap were performed. Principal Component Analysis (PCA) was performed with the software package MetaboAnalyst 3.0 using the default weighted covariance estimation functions [31]. The analysis was carried out in phloem sap of both scion and rootstock of all the nine rootstock/scion combinations, using a GC-MS based protocol for metabolite profiling. Statistical significance of GC-MS and UPLC-QTOF-MS/MS analyses was estimated by using two-way ANOVA (adjusted *P*<0.05).

## Results

### Effects of scion, rootstock and interaction on fruit yield, weight and quality

Table 1 presents fruit yield measurements of three successive years, showing an interaction between scion and rootstock. While Michal on Volka showed the highest yield, Merav and Orra on SB-812 and SO showed the lowest yield (Table 1). As for quality parameters, TSS, TA, their ratio and vitamin C levels were all affected by scion-rootstock interaction (Table 1). TSS was high for Michal and Orra on SO and Michal on SB-812, and low for Merav on SO and on Volka and for Orra on Volka, compared to other scion-rootstock combinations. TA in Merav was high for all three rootstocks and low in Michal and Orra on SB-812 and Volka. Accordingly, TSS/TA ratio was high in Michal on SB-812 and Volka and low in Merav, specifically on Volka. Vitamin C levels were low in Merav (for all rootstocks) and in Michal on Volka, and high in Orra on Volka.

**Table 1:** Mandarin fruit quality parameters analysis.

	ME/812	ME/SO	ME/V	MI/812	MI/SO	MI/V	O/812	O/SO	O/V	*P* scion	*P* rootstock	*p* interaction
**Yield (kg/tree)**	44.7±5.4cd	44.5±3.3cd	58.1±4.4c	102.1±4.7b	100.9±8.2b	152.2±7.8a	32.2±1.7d	44.2±2.7cd	65.9±2.2c	*	*	0.002
**TSS (%)**	14.8±0.2bcd	14.2±0.2de	12.9±0.4f	15.6±0.2ab	16.2±0.2a	15.2±0.1bc	14.6±0.2cd	15.6±0.1ab	13.6±0.1e	*	*	*
**TA (%)**	1.3±0.036a	1.29±0.05a	1.32±0.03a	0.92±0.03cd	1.04±0.03bc	0.86±0.02d	0.98±0.02bcd	1.07±0.01b	0.88±0.03d	*	*	0.002
**TSS/TA ratio**	11.3±0.21d	11.1±0.54d	9.5±0.15e	17.0±0.35ab	15.6±0.38bc	17.7±0.28a	15.0±0.31c	14.6±0.21c	15.6±0.51bc	*	0.06	*
**Ascorbic acid (mg/100 ml)**	31.3±10.5d	32.2±7.0d	32.0±7.9d	48.3±10.4b	39.7±14.8c	36.7±11.2cd	47.7±15.5b	50.1±17.8b	58.1±14.8a	*	0.17	*

TSS-total soluble solids; TA-Titrable acidity.

*p<0.0001

### Effect of scion/rootstock interaction on metabolic composition in fruit juice

**Fig 2:**
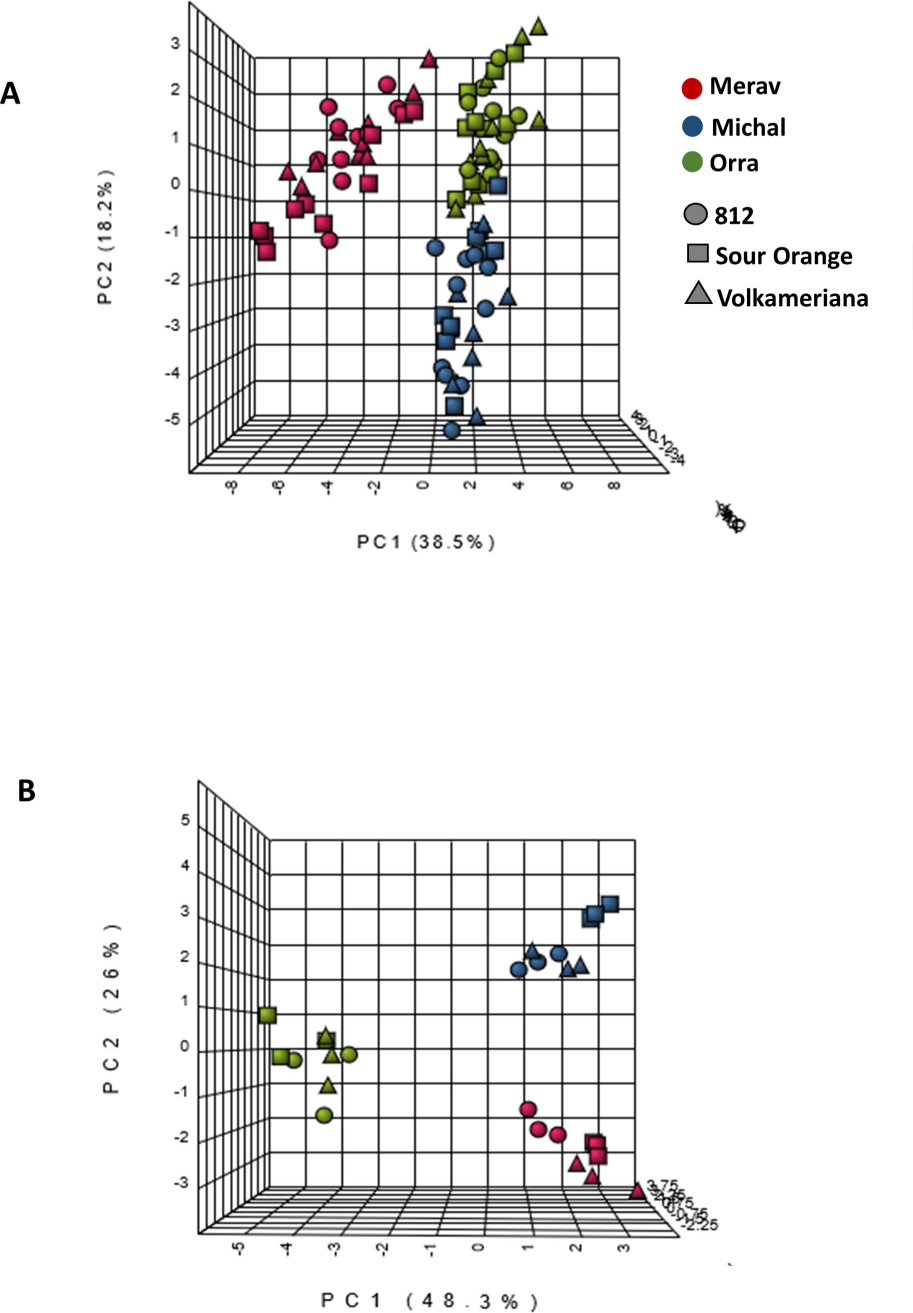
Principal component analysis of mandarin fruit juice from scion/rootstock combinations with 2-way ANOVA analysis. (A) primary metabolism (B) secondary metabolism.

In general, the rootstocks showed a direct effect on eight metabolites in the juice (‘directly’ refers to the metabolites affected only by rootstock and those affected by both scion and rootstock), and on another seven compounds in interaction with scion, total of 15 of 48 juice metabolites (Table 2). To determine the effect of scion/rootstock combinations on relative abundance of primary metabolites in mandarin juice, a GC-MS based protocol for profiling was employed. Thirty metabolites were annotated, out of which 24 metabolites were significantly related to the scion variety irrespective of their rootstocks (Table 2). Two metabolites were affected by both scion and rootstock, and one was differently affected by scion according rootstock (interaction effect). The rootstock direct effect was demonstrated on two metabolites: SB-812 was higher in proline and lower in quinic acid (S1 Table). In interaction with the scion, the rootstock affected one metabolite, threonic acid, which was high in Michal on Volka, lower in Michal on two other rootstocks and low in all other combinations. On a PCA analysis samples separated based on their scion (Fig 2A).

**Table 2:** Counts of primary and secondary metabolites for mandarin as a result of the scion, rootstock, both scion and rootstock, or the interaction between them. Rootstock direct- includes all metabolites that were affected directly by rootstock, including those under Rootstock and those under Rootstock and Scion.

	Rootstock	Scion	Rootstock and scion	Rootstock direct	Rootstock Interaction	Non- affected	Total annotated
**Juice GC**	0	24	2	2	1	3	30
**Juice LC**	0	5	6	6	6	1	18
**Juice Total**	0	29	8	8	7	4	48
**Scion sap**	1	4	5	6	42	1	53
**Rootstock sap**	6	8	8	14	33	0	55

For secondary metabolite profiling, we used a LC-MS-QTOF protocol. Eighteen metabolites were annotated, of which six were affected only by scion, five were affected by both scion and rootstock, and six metabolites changed among scions according to rootstock (interaction effect) (Table 2). In total, the rootstock directly affected six compounds: nomilin, nomilinic acid-17-beta-D-glucopyranoside and vicenin-2- low in SB-812; didymin and narirutin- lower in SB-812 than in Volka, limonin-higher in SB-812 than in SO (S2 Table). In interaction with the scion, the rootstock affected six metabolites: tangeritin- high in Michal on SO, rutin- high in Michal on SO and SB-812, diosmin- high in Orra on SO, tryptophan- high in Merav on SO and Volka, phenylalanine- high in Merav on SO and Volka and low in Michal on Volka and in Orra on SB-812 (S2 Table). A PCA analysis showed a scion-based separation (Fig 2B).

### Effect of scion, rootstock and interaction on scion phloem sap composition

**Fig 3:**
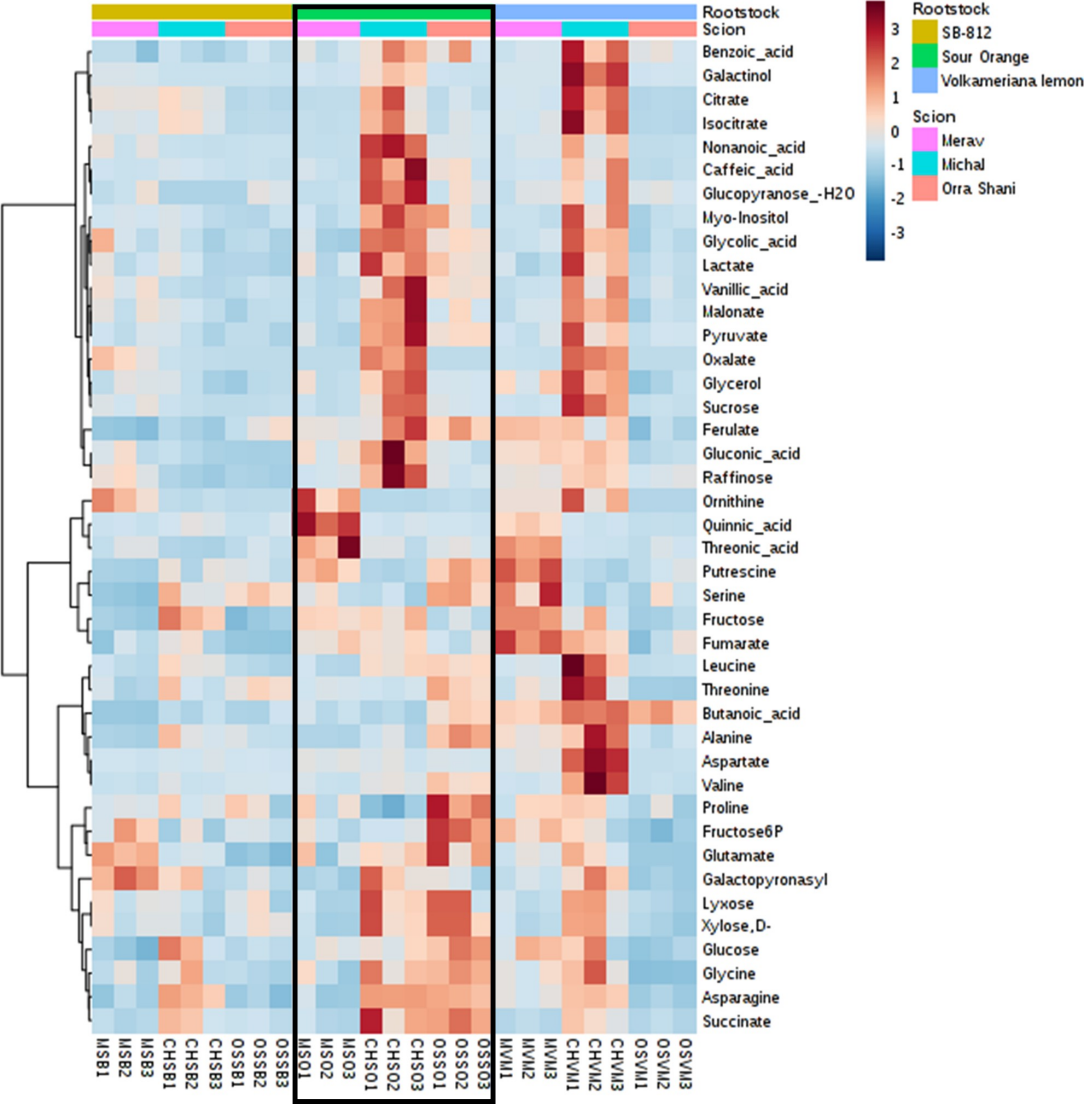
Heat map of scion-rootstock interactive metabolites in scion phloem sap.

A total of 53 metabolites were annotated in phloem sap of scions in all nine scion/rootstock combinations. In scion sap, one compound was affected only by rootstock, four only by scion, five by both rootstock and scion, and 42 changed in scion sap depending on specific scion/rootstock interaction (Table 2). Rootstock directly affected six metabolites: ribonic acid was highest in Volka, followed by SO and lowest in SB-812, phosphoric acid, pyroglutamate, malate and cysteine were low in SB-812, and maleate was high in Volka. Another 42 metabolites were affected by rootstock in interaction with the scion, for a total of 48 (S3 Table). The scion sap metabolites which were interactively affected by rootstock are shown in Fig 3, and include 11 sugars, 13 amino acids, six TCA/glycolysis metabolites, 10 organic acids and two lipid compounds (S3 Table). The proportions of these groups among the scion sap interactive metabolites are generally the same as in among the total scion sap metabolites.

### Effect of scion, rootstock and interaction on rootstock phloem sap composition

**Fig 4:**
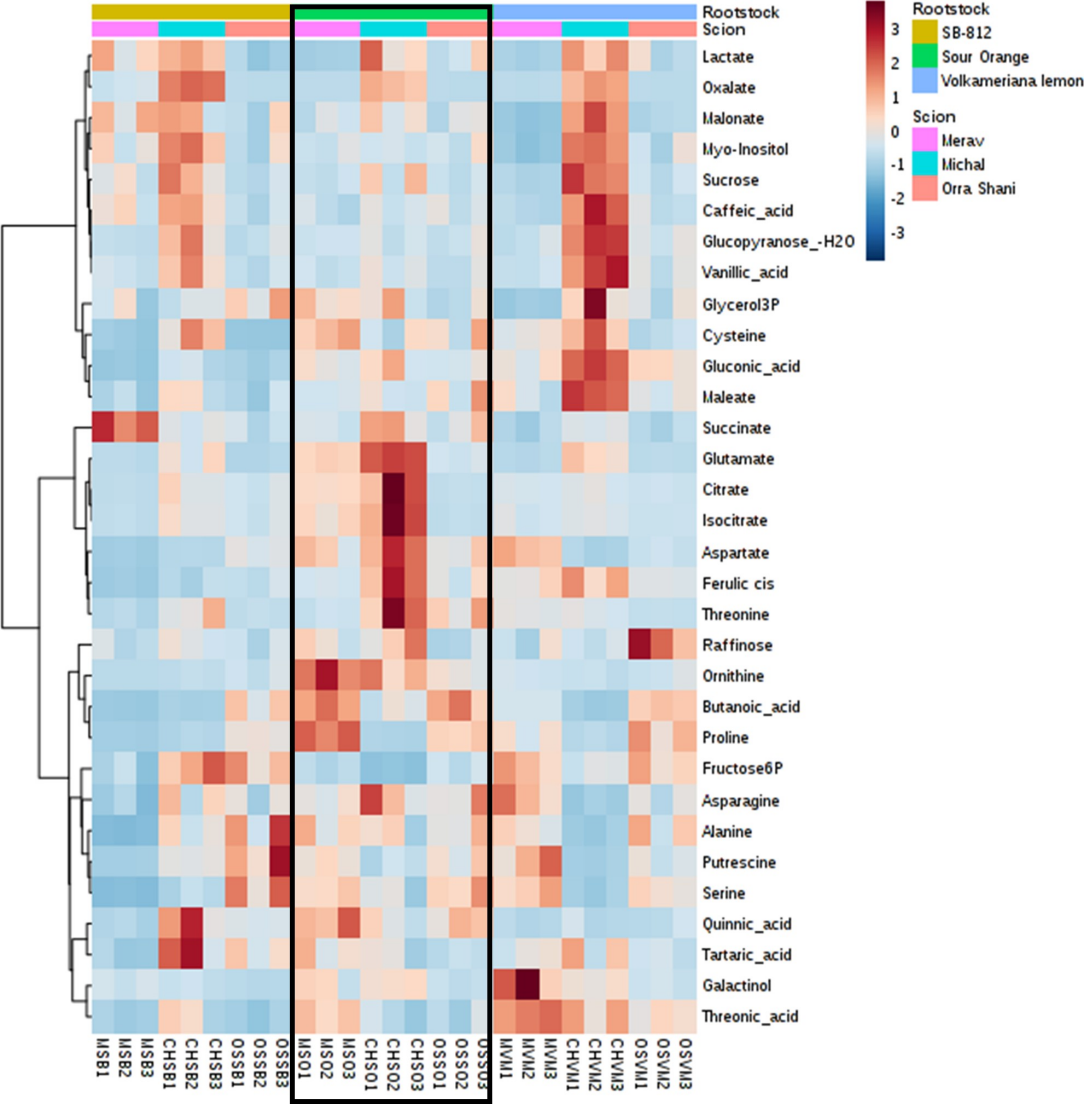
Heat map of scion-rootstock interactive metabolites in rootstock phloem sap.

A total of 55 metabolites were annotated in phloem sap of rootstocks in all nine scion/rootstock combinations. Six were affected only by rootstock, eight only by scion, eight by both rootstock and scion, and 33 changed in rootstock sap depending on specific scion/rootstock interaction (Table 2). In total, the rootstock directly affected 14 metabolites: leucine was higher in SO than in SB-812, valine, trans-ferulic acid, fructose and glucose were high in SO, glucose 6P, xylose, phosphoric acid and lyxose were low in Volka, malate, glycine and fumarate were low in SB-812, galactopyranosyl was high in Volka, and glycolic acid was higher in SB-812 than in Volka. In addition, rootstocks affected 33 more metabolites in interaction with the scion (S4 Table). The rootstock sap metabolites which were interactively affected by the rootstock are presented in Fig 4, and include six sugars, 11 amino acids, four TCA/glycolysis metabolites, 11 organic acids and one lipid (S4 Table). The proportions of these groups among the rootstock sap interactive metabolites are generally the same as in among the total rootstock sap metabolites, with the exception of lipids, which are 9% of total annotated compounds, but only 3% of the interactive.

## Discussion

In order to determine the scion-rootstock interaction in citrus trees, we studied their relation through analysis of fruit juice in addition to phloem sap samples from above and below the graft, as well as yield and chemical characteristic of the fruit of nine a grafted citrus combinations. Based on our results, we found that in addition to known effects of rootstocks on scion performances, scion and rootstock also interact to affect fruit juice and phloem sap composition.

Regarding rootstock effect on fruit yield, Volka enhanced the yield of Michal, and also of Orra compared to SB-812. At the same time, SB-812 and SO had low yields in combination with both Merav and Orra. However, no rootstock had a significant effect on yield in combination with Merav. Volkameriana lemon is considered to produce vigorous trees producing large quantities of fruits [32]. Earlier some studies from Florida [33] suggested that yields and net profits over the long term are higher for trees grafted on Volka than for others grafted on less vigorous rootstocks. Our results show that Volka's effect on yield is scion-dependent, and is more pronounced in some scions than in others.

The physio-chemical results strongly indicated that the effect of rootstock on mandarin fruit juice quality is rather a complex phenomenon that depends on a specific interaction between the rootstock and each scion variety. For instance: while Volka negatively affected TSS levels in Merav and Orra, it did not have such effect in Michal; while SO positively affected Michal and Orra TSS levels, it did not affect Merav; and while Volka induced TSS/TA ratio in Michal, the opposite was true for Merav. Additionally, Volka induced vitamin C contents in Orra, but hindered its production in Merav and Michal. Scion-rootstock interactions thus affect both fruit taste quality and its nutritional value.

Our results show that fruit juice composition was mainly affected by scion, as of 48 metabolites only 15 were affected by rootstock (eight directly and seven through scion interaction). Of these, eight were related to flavonoids and their biosynthesis: quinic acid, vicenin-2, didymin, narirutin, tangeritin, rutin, diosmin and the amino acid phenylalanine. Another four compounds were limonoid related (nomilin, Nomilinic acid-17-beta-D-glucopyranoside, limonin, Limonin-17-B-D-glucopyranoside), two were amino acids (proline and tryptophan) and one organic acid (threonic acid). The health-related effects of citrus flavonoids have been vastly reported, including anti-oxidative, anti-carcinogenic and anti-diabetic effects[34], and thus they are also marketed as nutritional supplements. Quinic acid is integral in shikimic acid metabolism [35], leading to the biosynthesis of flavonols and flavonoids [36]. Citrus limonoids are ascribed to fruit bitterness, but also possess favorable health properties including anti-oxidative, hypocholesterolemic and anti-carcinogenic effects [37].

By analyzing phloem sap composition above and below the grafting zone (scion phloem sap and rootstock phloem sap, respectively) we were able to learn about scion and rootstock interaction in grafted trees. As opposed to fruit juice, both scion sap and rootstock sap were chiefly affected by rootstock and by scion-rootstock interactions: rootstock affected 48 of 53 metabolites in scion sap (six directly and 42 through scion interactions), and 47 of 55 compounds in rootstock sap (14 directly and 33 through scion interactions). It is unclear whether the inconsistency between fruit juice and phloem sap results from a dilution effects occurring along the stem, different time of sampling or organ-dependent gene expression

When considering directly affected metabolites in the sap (from both scion and rootstock), results show that levels of all these metabolites were low in SB-812. These include ribonic acid, malate, fumarate, phosphoric acid, cysteine, glycine, and pyroglutamate. Juice directly affected metabolites followed the same trend of low levels in SB-812, including quinic acid, nomilin, nomilinic acid-17-beta-D-glucopyranoside and vicenin-2. Additionally, the levels of interactively affected scion sap compounds are also lower in SB-812 in comparison to both other rootstocks, irrespective of the scion, and with not much interaction with the scion grafted (Fig 3). Rootstock sap interactively affected metabolites also generally followed this trend of low levels in SB-812 (Fig 4). Proline was the only directly-affected metabolite which levels were higher in SB-812 compared to SO and Volka, and may indicate that as a rootstock SB-812 generates pronounced stress conditions in the trees, as proline was previously reported to be involved in plant stress response [38]. This stress might by resulting from low levels of essential metabolites, as shown for SB-812 in fruit juice, scion sap and rootstock sap. The lower content of amino acids and sugars in the sap of SB-812, possibly reflective of lower N assimilation and C/N translocation, could eventually lead to a lower quality of fruits. For example, as shown above, SB-812 showed the lowest levels for limonoids nomilin and nomilinic acid-17-beta-D-glucopyranoside. The metabolism of nomilin, the precursor for limonoids, starts in the phloem region via terpenoid biosynthetic pathways from acetate and mevalonate, via farnesyl pyrophosphate [39]. These pathways depend upon glycolytic precursors. It is thus tempting to suggest that a lower malate and fumarate content in the sap of SB-812 would be associated to lower nomilin in the juice.

In contrast to SB-812, SO rootstock had high levels of all directly affected metabolites, in both scion and rootstock sap, including valine, ferulic acid, fructose, and glucose. Volka rootstock showed a mixed trend, with low levels of glucose 6P, xylose lyxose and phosphoric acid, and high levels of ribonic acid, maleate, malate and galactopyranose. Both rootstocks demonstrated much interaction with the scion, presenting different profiles for each rootstock-scion combination. Specifically such combinations can be classified according to the degree of scion-rootstock of interaction, manifested by high levels of metabolites (number and intensity). Metabolically strong rootstock-scion interaction is favorable, as a more interactive scion-rootstock system is presumably better in means of plant physiological state, reflecting a more fluent connection between the two organisms of the system. As such, it enables a reciprocal and coherent communication, expressed as metabolite transfer between the two parts, and possibly allowing more flexibility in response to environmental conditions, including stress, resulting in long-lasting trees.

Based on that parameter, it is evident in scion sap that the Orra-Volka combination has the lowest interaction (aside from all SB-812 combinations as discussed above), followed by Merav-SO, Merav-Volka and Orra-SO. The Michal-SO and Michal-Volka are the best combinations in regard to rootstock-scion interaction. In rootstock sap (Fig 4) it can be seen that in general, the interaction is more significant (shown by number of high-level compounds and by fold change (FC) ratio), showing even some level of scion-rootstock interaction for certain SB-812 combinations. Rootstock sap combinations follow the trend shown in scion sap, with the best performance to Michal-Volka and Michal-SO, and lowest interaction in 812, mainly for Merav and Orra.

Although the number of interactive metabolites is higher in scion sap than in rootstock sap (47 vs.33), the degree of interaction is greater in the rootstock sap, while metabolites in the scion sap are less affected. Together with the larger number of directly affected metabolites in rootstock sap (14) compared to scion sap (6) and juice (8) (while total number of compounds is generally the same), this suggests a distance-dependent effect for the rootstock. The rootstock imposes its effect locally, and thus it is greatest in rootstock sap, followed by scion sap, and lowest in juice, as shown by the number of directly and interactively affected compounds, and the degree of influence.

Notably, among interactive metabolites are important transporters of carbon, such as sucrose and raffinose [40] and of nitrogen, like 4-amino butanoic acid (GABA) and glutamate [41], which showed significant change in their relative content. These data suggest that selection of an appropriate rootstock may provide a powerful means to manage the plant energy metabolism and transport for the benefit of the growth and fruiting of the scion.

Interestingly, a small number of metabolites were affected by neither scion, rootstock or their interaction. In juice these were apigenin, myo-inositol, sucrose and asparagine, and tartaric acid in scion sap. These compounds might play an important role in citrus, as suggested for tartaric acid in citrus as a source of ascorbic acid [42] or for myo-inositol for cell signaling, mainly under stress [43], thus deliberately maintained at constant levels by the tree. Alternatively, they could be affected by different factors, other than scion or rootstock.

Taken together, our results clearly suggest that rootstock-scion combination is a reciprocal system, crucial to fruit quality and juice and sap composition, with possible impact over tree physiology. Our work suggests a reciprocal relationship between the scion and roots in a grafted tree, whereby a mutual alteration/acclimation process in the metabolism is developing in the forced connection between two different genotypes. Furthermore, characteristics of either a scion or a rootstock cannot be determined for themselves, but rather only in interaction with each scion/ rootstock. Accordingly, choosing a rootstock-scion combination should first take into consideration the required characteristics of either the scion or the rootstock (e.g., salinity tolerance), followed by a careful choice of its compatible partner.

Specifically, our results indicate that SB-812 has the lowest level of scion-rootstock interaction, while Michal-Volka, Michal-SO and Orra-SO combinations have the highest level of interaction. Further research is required in order to determine the biochemical mechanisms underlie this phenomenon, and should include a more detailed analysis of the interaction, including tree-physiological parameters, in addition to possibly identifying specific chemical and physiological markers to indicate the quality of interaction, in order to examine new combinations. Additionally, to better understand the locality attribute of scion-rootstock interaction, more sap measurements should be done alongside the grafting zone, in varying distances.

Principally, every rootstock- scion combination is kind of a new organiam by itself. As a result, all the metabolic pathways are potentially affected by the grafing in both rootstock and scion. Yet, some of the metabolic pathways are strongly affected by rootstock (branced chain amino acid metabolism) while others by scion (fatty acid metaolism and secondary metabolites metabolism). This makes sense since its well known that some of the hormones that move along the plant are dominantly produced by the roots (e.g. cytokinins) while other are produced by the shoots (e.g. auxin). Therefore whenever we choose to create a new rootstock-scion combination, its potential effect on metabolic pathways should be taken into consideration. The effect on the pathways may vary along season and based on tree's age. The scion-rootstock union unite intimately, providing a viable system for the uptake and translocation of minerals, water, assimilates, and hormones throughout the entire lifespan of the tree [44–46]. Improved understanding of this crosstalk will enhance the selection process for grafting material, favoring combinations that will benefit fruit quality, fruit metabolism and phloem sap composition.

## Conclusion

In summary, in this work we show for the first time a reciprocal interaction between rootstock and scion. Some of the rootstock effect that was found on the rootstock phloem sap also appeared in scion phloem sap, but not in fruit. Such inconsistency between the phloem sap and fruit might be due to the distance from the grafting zone (a dilution effect with distance), to sampling timing, or could arise from organ-dependent gene expression. The distance, organ and timing effects are currently investigated.

## Supporting information

S1 Table Primary metabolites identified in mandarin fruit juice in scion-rootstock combinatios. Small letters- scion statistics; Capital letters-rootstock statistics; bold letters-interaction statistics.
(XLSX)

S2 Table Secondary metabolites identified in mandarin fruit juice in scion-rootstock combinatios. Small letters- scion statistics; Capital letters-rootstock statistics; bold letters-interaction statistics.
(XLSX)

S3 Table Primary metabolites identified in mandarin scion phloem sap. Small letters- scion statistics; Capital letters-rootstock statistics. FC-fold change.
(XLSX)

S4 Table Primary metabolites identified in mandarin rootstock phloem sap. Small letters- scion statistics; Capital letters-rootstock statistics. FC-fold change.
(XLSX)
